# Assessment of Antimicrobial Activity of Pomegranate, Cranberry, and Black Chokeberry Extracts against Foodborne Pathogens

**DOI:** 10.3390/foods10030486

**Published:** 2021-02-24

**Authors:** Maria Daoutidou, Stavros Plessas, Athanasios Alexopoulos, Ioanna Mantzourani

**Affiliations:** 1 Laboratory of Food Processing, Faculty of Agriculture Development, Democritus University of Thrace, 68200 Orestiada, Greece; maridaou1@agro.duth.gr (M.D.); splessas@agro.duth.gr (S.P.); 2 Laboratory of Microbiology, Biotechnology and Hygiene, Faculty of Agriculture Development, Democritus University of Thrace, 68200 Orestiada, Greece; alexopo@agro.duth.gr

**Keywords:** plant extracts, natural preservatives, *Staphylococcus aureus*, *Escherichia coli*, *Streptococcus pyogenes*, pork meat

## Abstract

Herbal and plant extracts are being applied to a wide range of foods against different types of foodborne pathogens. In the present study, ethanolic and aqueous extracts of different concentrations (5% *v/v*, 10% *v/v*, and 20% *v/v*) from cranberry (*Vaccinium macrocarpon*), black chokeberry (*Aronia melanocarpa*), and pomegranate (*Punica granatum* L.) plants were applied in five concentrations (62.5 to 1000 mg/mL) against foodborne strains of *Staphylococcus aureus*, *Escherichia coli*, and *Streptococcus pyogenes*. The results revealed that a low concentration of solvents (5% *v/v*) did not exhibit decreased antimicrobial activity in comparison with higher solvent concentrations (10% and 20% *v/v*). Additionally, both aqueous and ethanolic extracts were highly effective against pathogens even in their low concentrations (62.5 mg/mL and 125 mg/mL). Likewise, the extracts exhibited promising results (aqueous extracts of pomegranate, cranberry, and black chokeberry in a food-compatible concentration of 2% *w/v*) were applied to raw pork meatball production, and their antimicrobial activity was recorded versus *Enterobacteriaceae*, total mesophilic bacteria (TMB), lactic acid bacteria (LAB), *Staphylococcus* spp., *Pseudomonas* spp., and yeasts/molds. The outcome demonstrated that meatballs that contained aqueous extracts of pomegranate were more resistant to spoilage compared to all of the other samples, as it was preserved for more days. Likewise, these extracts of a plant origin could be used as natural preservatives in meat products, even in their low concentrations.

## 1. Introduction

Natural preservatives, especially herb or plant extracts, are suitable for application in food products [1]. They are acceptable by consumers and, simultaneously, they provide health benefits, such as antioxidant and antimicrobial activity. Therefore, such natural extracts might be used as multifunctional preservatives in several cases of food products, exhibiting desirable technological characteristics, like prolonged shelf life and ameliorated properties.

Among numerous plant origin materials, berries are known as the richest sources of polyphenolic antioxidants, mainly associated with antimicrobial activity [2]. More specifically, black chokeberry (*Aronia melanocarpa*) fruits exhibit remarkable antioxidant activity, mostly because of their high polyphenol content [3]. They belong to the *Rosaceae* family. As secondary metabolites, their chemical composition includes anthocyanins, proanthocyanidins, and hydroxycinnamic acids, quercetin and its glycosides, and epicatechin [4,5]. The main health benefits of black chokeberries are antidiabetic, antimutagenic, antioxidative, and cardioprotective properties [4]. The same data are available for their acetonic extracts as well. Black chokeberry extract exhibits strong antimicrobial properties against a broad spectrum of microorganisms (*E. coli*, *Salmonella* spp., *S. aureus*, *L. monocytogenes*, *Pr. vulgaris*, *P. aeruginosa*, *Kl. pneumoniae*) [6].

Furthermore, cranberry (*Vaccinium macrocarpon*) is a berry fruit that has been consumed for centuries. It belongs to the *Ericaceae* family. A significant advantage of its consumption is its antibacterial and antifungal activity against numerous illness-causing pathogenic bacteria [7,8], including *Staphylococcus aureus*, *Escherichia coli*, and *Streptococcus* spp. Especially, the American cranberry is known for its activity against urinary tract infections, dental decay, stomach ulcers, and cancers [9]. This activity has been explained by its low pH and by its bioactive compounds [10]. These compounds include phenolic acids, tannins, proanthocyanidins, and flavonoids (anthocyanins and flavonols) [11,12,13].

On the other hand, pomegranate (*Punica granatum* L.) is characterized by high antioxidant activity, while tannins are mainly responsible for observed antimicrobial properties [14]. The plant belongs to the *Punicaceae* family, and grows in temperate climate areas (SE Asia, the Mediterranean, the United States, and elsewhere). Its peels have been used for centuries in traditional medicine as a remedy for diarrhea and dysentery [15]. Likewise, the antimicrobial activity of juice extracts from pomegranate peel flour has been evaluated against several pathogenic bacteria, like *Pseudomonas aeruginosa*, *Escherichia coli*, *Listeria monocytogenes*, *Salmonella* spp., and *Staphylococcus aureus* with satisfactory results [16].

From the literature, it is also known that several functional ingredients are present in cranberries and can be extracted, like flavonols and hydroxycinnamic and other organic acids, usually called phytochemicals, that have bacteriostatic or bactericidal activity against foodborne or clinical pathogens [17,18]. It depends on the polarity of the solvent what kind of phytochemicals will be extracted. Ethanol and methanol mainly solve non-polar ingredients, and water solves the polar ones [19,20]. The combined use of water and organic solvents typically facilitates the extraction of chemical compounds soluble in water and organic solvents. Nevertheless, the extraction method, temperature, length of exposure to the solvent, differences in quantity, and profile of phenolic compounds in specific fruit types should be taken into consideration [21].

To this direction, fruit phenolic-rich extracts may serve as antimicrobial agents against pathogenic bacteria of food or clinical origin. Polyphenols, tannins, and flavonoids have gained a lot of attention from the scientific community, mostly as a resource for the production of new products, like biopreservatives or natural preservatives in a variety of food products, beverages, meat products, etc., where they could replace the common synthetic preservatives. The growing awareness of the consuming public has launched research into this field lately [22].

The objective of this study was to examine the antimicrobial potential of two different types of extracts (ethanolic EtOH and aqueous) from three fruits with high phenolic content (black chokeberry, cranberry, and pomegranate) against three foodborne pathogens (*E. coli*, *S. aureus*, and *S. pyogenes*). Furthermore, the aqueous extracts of the three fruit juices were selected and tested as natural preservatives (antimicrobial capacity) in raw pork meatball (2% *w/v*) production, mostly because of their effectiveness and their compatibility for food consumption. The microbial groups examined include *Enterobacteriaceae*, lactic acid bacteria (LAB), total mesophilic bacteria (TMB), yeasts and molds, *Staphylococcus* spp., and *Pseudomonas* spp.

## 2. Materials and Methods

### 2.1. Raw Material

Fresh cranberries (*Vaccinium macrocarpon*), pomegranates (*Punica granatum *L.), and black chokeberries (*Aronia melanocarpa*) were used for the preparation of the respective extracts applied to determine the growth inhibition. These samples were selected from the local market of Nea Orestiada in northeastern Greece. All fruits were produced from local farms (conventional) situated at a distance of 60 km from the above city. The climate conditions in the area are typical of the Mediterranean, with moderate cold during the winter and heat during the summer season. The mean annual conditions in the region are: temperature 15.2 ± 7.8 °C, precipitation 45.4 ± 22.9 mm, and relative humidity 67.6 ± 8.8%. The harvest period for all of the three fruits examined is during autumn, from September through November.

### 2.2. Preparation of Bacterial Strains

*Escherichia coli* (strains 7 and 1303), *S. aureus* (strains 8 and 22), and *S. pyogenes* (strains 29 and 1143) were isolated and characterized at a previous time in our laboratory via Viatek 2 Compact (Biomerieux, Marcy-l’Etoile, France). All of them were kept frozen in Tryptone Soya Broth (Himedia Laboratories Pvt, Ltd., Mumbai, India) and enriched with 30% glycerol until use. *S. aureus* strains were non-methicillin resistant, and *E. coli* strains were non-extended spectrum β-lactamase producers (latex agglutination test: Oxoid^TM^ Ltd., Hampshire, UK and CLSI: Clinical and Laboratory Standards Institute double disk method) [23].

Before application, all strains were incubated in the appropriate conditions to ensure optimal growth. For *S. aureus* cultures, mannitol agar was used, for *E. coli* cultures, TBX agar was used, and for *Streptococcus* cultures, blood agar was applied (Himedia Laboratories Pvt, Ltd., Mumbai, India).

### 2.3. Preparation of Extracts

The extraction method was an adaption from the study of Yang, Jia, and Zu [24]. Beads from each fruit were blended for 1 min at room temperature in a stomacher apparatus. Ten grams of each puree were mixed with 200 mL of each solvent (5% *v/v*) at 60 °C in an Erlenmeyer flask with agitation for two hours. Respective preparations were followed for 10% and 20% *v/v*.

The aqueous solutions were filtered through a 0.45 mm filter paper under vacuum, and filtrates were centrifuged at 5000× *g* for 20 min at room temperature. The supernatants were evaporated at 5 mL and at 50 °C in an evaporator (Model R204B3, Senco Technology Ltd., Shanghai, China). In the concentrated solutions, 5 mL of distilled water was added (initial concentration: 2 g/mL). The tested extract concentrations varied from 62.5, 25, 250, 500, and 1000 mg/mL. The samples were kept at −4 °C.

### 2.4. Determination of Antimicrobial Activity

Mueller−Hinton agar (Himedia Laboratories Pvt, Ltd., Mumbai, India) was the agar selected for the antibiograms. The initial inoculation level reached approximately 150 × 10^6^ CFU/mL (colony-forming units) (0.5 McFarland scale) for all strains tested.

The method applied was the agar diffusion method. Briefly, 1 mL of inoculum from each strain was plated on the surface of Mueller−Hinton agar, and the antibiotic discs were placed as well.

Levofloxacin (5.03 BCg/disc) for *E. coli*, penicillin-G (10 units/disc) for *S. aureus* strains, and vancomycin (30 μg/disc) for *Streptococcus* strains were employed as reference antibiotics; 10 μL of each extract were placed on sterile discs (Himedia Laboratories Pvt, Ltd., Mumbai, India), and petri dishes were incubated overnight at 37 °C. The inhibition diameter around the disc was measured with a Traceable Carbon Fiber Digital Caliper (Fisher Scientific Ltd., Nepean, ON, Canada). All measurements were performed in triplicate (*n* = 3).

### 2.5. Preparation of Raw Meatballs

Raw meatballs were prepared according to traditional techniques and recipes. A batch consisting of ground pork meat (2 kg), lard (0.5 kg), sodium chloride (1.5% *w*/*w*, 3 g), and black pepper (0.2 g) was produced. The mixture was well-homogenized, and four different types of meatballs were prepared, namely: (a) control samples (C) where no extract was used, (b) cranberry aqueous extracts (WC), (c) aronia aqueous extract (WA), and (d) pomegranate aqueous extract (WP), with a concentration of 5% *v/v* in the cranberry, black chokeberry, and pomegranate respectively added (all in a concentration of 2% *w/v*). Meatball samples were kept at 4 °C in polystyrene trays covered with protective film for the entire study period. All experiments were carried out in triplicate. Samples from each treatment were collected at various intervals (first, third, fifth, eight, and twelfth day) and subjected to chemical and microbiological analysis. Data after the eight day of storage are not shown, since the spoilage of the meatballs was obvious in odor and sight.

### 2.6. Microbiological Analysis of Meatball Portions

The samples were subjected to microbiological analysis to monitor the dynamic changes in the population of microorganisms responsible for the spoilage of the meatballs (*Enterobacteriaceae*, total mesophilic bacteria (TMB), yeasts and molds, *Staphylococcus* spp., and *Pseudomonas* spp.). In addition, the levels of LAB were also determined. Therefore, representative 25 g portions of meatball samples were taken from the interior, blended with 225 mL of sterilized 1/4 Ringers solution (Sigma-Aldrich, Saint Louis, MO, USA), and subjected to serial dilutions.

The following tests were performed: (i) total aerobic counts on plate count agar (Oxoid Ltd., Hampshire, UK) at 30 °C for 48 h, (ii) lactobacilli (Gram (+), catalase (−)) on acidified MRS agar (Oxoid Ltd., Hampshire, UK) at 37 °C for 48 h anaerobically (anaerobic jar, Anerocult C, Merck, Taufkirchen, Germany), (iii) enterobacteria on violet red bile glucose agar (Oxoid Ltd., Hampshire, UK) at 37 °C for 24 h, (iv) pseudomonads on pseudomonas CFC selective agar (Merck, Taufkirchen, Germany) at 30 °C for 72 h, (v) Staphylococci on a Baird Parker egg yolk tellurite medium (Oxoid Ltd.,Hampshire, UK) at 37 °C for 48 h and confirmed by a positive coagulase test, and (vi) yeasts and molds on malt agar (Oxoid Ltd., Hampshire, UK) (pH was adjusted to 4.5 by a sterile solution of 10% lactic acid) at 30 °C for 48 h. All incubations were further extended up to 120 h, but no extra colonies were observed. Gram staining and catalase tests were performed for LAB confirmation. Results are presented as log CFU (mean colony-forming units) on solid media culture plates containing between 30 and 300 colonies per gram of meatball.

### 2.7. pH Measurement

The pH values of the meatballs were estimated as proposed by the FDA. In brief, 10 g of the samples were homogenized in 50 mL of deionized water, and the measurement was performed with an Mi 150 pH/Temperature Bench Meter (Martini Instruments, New York, NY, USA).

### 2.8. Statistical Analysis

The mean ± standard deviation was calculated for the zones of inhibition. Differences in the mean zones of inhibition for various strains, fruit extracts, and types of solvent were estimated by using the ANOVA procedure with *post hoc* comparisons. Pearson product moment coefficients were used to test correlations between the zones of inhibition and concentration of the fruit extracts or solvents. Bacterial counts were logarithmically transformed, checked for normality, and presented as log CFU/g ± standard deviation. Differences in bacterial counts were also checked by ANOVA with Tukey’s HSD *post hoc* application. Analysis was performed with SPSS v20^®^ (IBM Corp., Armonk, NY, USA) at a 95% significance level.

## 3. Results

The pH value of the samples was adjusted to 7.0 in order to ensure that the results recorded were not due to the natural acidity of the fruits [10]. Three different concentrations of two solvents were applied (5%, 10%, and 20% *v/v*) for each fruit. The respective zones of inhibition (ZOIs) were measured (in mm), and the results are shown in the following Table 1, Table 2 and Table 3. At this point, it should be mentioned that, in the case of levofloxacin (5 μg/disc), the *E. coli* strains were characterized as sensitive when the diameter of the ZOI was larger than 25 mm and resistant when the ZOI was smaller [25]. For vancomycin (30 μg/disc) and *Streptococcus pyogenes* strains, the respective cutoff diameter was 17 mm. For larger ZOIs, the strains were characterized as sensitive, and for smaller diameters were characterized as resistant [26]. Finally, in the case of *S. aureus* strains, where penicillin (10 units/disc) was applied, when the diameter of the ZOI was larger than 37 mm, the strains were characterized as sensitive, and for a diameter of ZOI smaller than 26 mm, the strains were characterized as resistant [27]. Overall, there were no mean inhibition zones below 7 mm in diameter, and therefore, no negative results were recorded in any of the experiments.

### 3.1. Application of Cranberry Extracts

In Table 1, the results in the case of cranberry extract against the examined six foodborne pathogens are shown. On half of the strains tested, *E. coli* 1303 (F = 38.57, *p* < 0.05), *S. aureus* 8 (F = 48.15, *p* < 0.05), and *S. aureus* 22 (F = 34.17, *p* < 0.05), the zones of inhibition produced by the aqueous extract were larger than those from the ethanolic extract, indicating increased efficiency. 

Regarding the case of *E. coli* 7, the application of 20% *v/v* of aqueous extract in a concentration (E.C.) of 250 mg/mL provided a ZOI of 12 mm. Additionally, 5% *v/v* ethanol in an extract concentration of 125 mg/mL provided a zone with a diameter of 11 mm. Based on CLSI guidelines, *E. coli* 7 can be characterized as resistant, with levofloxacin as the antibiotic reference.

Regarding *E. coli* 1303, both solvents exhibited quite satisfying results. Nevertheless, 20% *v/v* of aqueous extract in a dose of 500 mg/mL revealed a larger zone of inhibition (13 mm). In ethanolic extractions, the solvent concentration of 5% *v/v* exhibited the best results in the lowest doses (125 mg/mL, 250 mg/mL, and 250 mg/mL, respectively). Based on CLSI guidelines, *E. coli* 1303 could be characterized as resistant.

In the case of *S. aureus* strains, *S. aureus* 22 was found to be more sensitive, with 5% *v/v* solvent concentration in every solvent. The respective E.C. was 62.5 mg/mL, 62.5 mg/mL, 62.5 mg/mL, and 125 mg/mL for ethanolic and aqueous extracts, respectively. The largest diameter (11 mm) was observed in the case of 10% water as the solvent with an E.C. of 125 mg/mL. 

*S. aureus* 8 had clearly encouraging results in the case of the aqueous extract, where a ZOI of 10 mm (with an E.C of 250 mg/mL) was measured in the cases of 10% *v/v* and 20% *v/v* as the solvent concentrations. 

In *S. pyogenes* 29, when water was examined as the solvent in the concentration of 5% *v/v*, 10% *v/v*, and 20% *v/v*, it exhibited a ZOI of 12 mm at an E.C. of 62.5 mg/mL, 250 mg/mL, and 250 mg/mL, respectively. *Streptococcus pyogenes* 1143 was a resistant strain according to CLSI, in contrast with *Streptococcus pyogenes* 29, which was sensitive. Nevertheless, a 5% *v/v* solvent concentration in each solvent provided promising results: 14 mm and 13 mm for ethanolic and aqueous extracts with an E.C. of 62.5 mg/mL and 62.5 mg/mL, respectively. In most of the experiments concerning *Streptococcus pyogenes* 29, ZOIs with diameters over 11 mm were recorded in every solvent concentration, while the vancomycin ZOI was 15 mm.

### 3.2. Application of Black Chokeberry Extracts

In Table 2, the results for black chokeberries are shown. Similar to cranberry, the zones of inhibition produced by the aqueous extract were larger than those from the ethanolic extract, indicating an increased efficiency. However, statistically significant differences were observed for strains *E. coli* 1303 (F = 9.45, *p* < 0.05), *S. aureus* 8 (F = 5.99, *p* < 0.05), *S. aureus* 22 (F = 50.17, *p* < 0.05), and *S. pyogenes* 1143 (F = 18.8, *p* < 0.05).

Regarding *E. coli* 7, the greater zone diameter was measured in the case of a 10% aqueous extract, approximately 10 mm (an E.C. of 250 mg/mL). Ethanolic extracts exhibited a ZOI of 8 mm, independently of the solvent concentration.

*E. coli* 1303 exhibited the best results in the case of 10% *v/v* of aqueous extraction (an E.C. of 500 mg/mL), with a ZOI of 13 mm. In the case of the ethanolic extract, 11 mm was the larger zone observed when a 5% solvent concentration was applied (an E.C. of 125 mg/mL). The respective diameter of the ZOI of the antibiotic tested, levofloxacin, was 31.7 mm.

*S. aureus* 22 provided the highest values of ZOIs in the case of 10% *v/v* and 20% *v/v* of aqueous extract, where the diameter was 11 mm, the same as the used antibiotic (penicillin), with an E.C. of 125 mg/mL and 250 mg/mL, respectively; 5% *v/v* of water provided zones of 10 mm (an E.C. of 62.5 mg/mL).

*S. aureus* 8 exhibited a really impressive result. The same moment penicillin provided a ZOI with a diameter of 10 mm, 10% *v/v* and 20% *v/v* of water gave a ZOI of 10 mm (equal to penicillin) with an extract concentration of 250 mg/mL for both of them. Quite satisfying results were recorded for both solvents in all of their concentrations.

Regarding *S. pyogenes* 29, water as the solvent in all of its concentrations (5%, 10%, and 20% *v/v*) exhibited zones with diameters of 13 mm and 12 mm, with an E.C. ranging from 62.5 mg/mL to 250 mg/mL. The same moment, vancomycin, the reference antibiotic, exhibited a zone with a diameter of 11 mm. Ethanol revealed satisfying results, with a ZOI of 12 mm and 13 mm for 5% *v/v* and 20% *v/v* ethanol concentrations, respectively (an E.C of 125–1000 mg/mL).

Regarding *S. pyogenes* 1143, water provided the most promising results as the solvent in all of its concentrations (5%, 10%, and 20% *v/v*), with ZOIs reaching the values of 13 mm, 13 mm, and 12 mm, and an E.C. of 62.5 mg/mL, 125 mg/mL, 250 mg/mL, respectively. It is noteworthy that the ethanolic extracts recorded good scores; the smallest diameter among the zones of inhibition was the value of 10 mm (5%), and the largest was 12 mm (10%). Vancomycin exhibited a diameter of 15 mm.

### 3.3. Application of Pomegranate Extracts

Finally, pomegranate and its extracts were the last to be examined against these bacterial strains (Table 3). In contrast to cranberry and black chokeberry extracts, ethanolic pomegranate extract appeared efficient against *E. coli* 1303 (F = 220.38, *p* < 0.05) and *S. aureus* 8 (F = 11.64, *p* < 0.05).

The results showed that, against *E. coli* 7, the most satisfying results were recorded when 20% *v/v* of water was used. A ZOI of 12 mm was then formed with an E.C. of 250 mg/mL. Levofloxacin provided a ZOI of 32.3 mm. Ethanolic extracts in all of their concentrations exhibited a ZOI of 10 mm with an E.C. of 250–1000 mg/mL.

Slightly different behavior was observed in the case of *E. coli* 1303. Here, ethanolic extracts with 5% and 20% showed zones of 11 mm. All of the other results reached a ZOI of 10 mm and 9 mm; meanwhile, levofloxacin showed a ZOI of 31.7 mm against this pathogen.

In addition, *S. aureus* 22 revealed the most promising results when 5% *v/v* of ethanol was applied, with a ZOI of 15 mm and an E.C. of 500 mg/mL, and with the application of 20% *v/v* of ethanol, a zone of 14 mm was observed (an E.C. of 1000 mg/mL). Finally, 20% *v/v* of water gave a ZOI of 14 mm (an E.C. of 1000 mg/mL). The ZOI against penicillin was 12 mm.

Regarding *S. aureus* 8, it was found that two cases of solvents provided larger ZOIs in comparison with penicillin, which had a ZOI of 10 mm against the particular strain. That was the case of 10% *v/v* ethanol (an E.C. of 500 mg/mL), with a ZOI of 12 mm. All of the aqueous extracts in all of their concentrations provided a ZOI of 8 mm. 

In conclusion, *S. pyogenes* 29, when tested, revealed that 20% *v/v* water (an E.C of 500 mg/mL), 10% *v/v* ethanol (an E.C. of 250 mg/mL), and 20% *v/v* ethanol (an E.C. of 250 mg/mL) gave a ZOI of 11 mm. The rest were found to have smaller ZOIs, including vancomycin, with a ZOI of 10 mm.

When *S. pyogenes* 1143 was examined, a ZOI of 14 mm was recorded in the case of 5% *v/v* ethanol (an E.C. of 62.5 mg/mL) and a ZOI of 13 mm when 5% *v/v* of aqueous extract was applied (an E.C. of 62.5 mg/mL). Vancomycin gave a ZOI of 14 mm, and it must be underlined that the other solvent concentrations gave better or almost equal ZOIs compared to vancomycin, revealing promising potential against this pathogen. The relative results are shown in Table 3.

### 3.4. Effectiveness of the Solvent Type per Strain

From the statistical analysis of the results as far as the effectiveness (inhibition zone in mm) of the solvent type per strain is concerned, it was revealed that, in the case of cranberry, aqueous extracts had the best performance against the three types of strains tested, with only one exception of *E. coli* 7. Results are shown in Table 4.

In the case of black chokeberry, *E. coli*, *S. aureus*, and *S. pyogenes* strains were more susceptible to aqueous extracts compared to their ethanolic counterparts. Results are shown in Table 4.

Finally, when pomegranate extracts were examined, results were basically differentiated. The *E. coli* 7 strain exhibited greater susceptibility to aqueous extracts, and, at the same moment, *E. coli* 1303 exhibited greater susceptibility to ethanolic extracts. *S. aureus* strains were more sensitive to ethanolic extracts, and *S. pyogenes* strains to ethanolic and aqueous extracts, with small differences. Results are shown in Table 4.

### 3.5. Effectiveness of Solvent Concentration

The results revealed that, out of 36 experiments, there were five positive and statistically significant correlations indicating an increase in effectiveness due to the increase of solvent concentration. On the contrary, there were five negative (statistically significant) correlations indicating a decrease in effectiveness due to the increase of solvent concentration. Nevertheless, most negative correlations were observed when ethanol was used as a solvent against *S. pyogenes* and *S. aureus* strains, while water gave the most positive correlations regardless of the strain. The respective results are shown in Table 5.

### 3.6. Effectiveness of Extract Concentration

The final factor examined was the effectiveness (ZOI) of the extract concentration against the different types of pathogens. The results pointed out that, with the exception of two cases (*E. coli* 1303, black chokeberry and *S. aureus* 8, pomegranate), multiple comparisons revealed that the various extracts were highly effective against pathogens even in their lowest concentration (62.5 mg/mL or 125 mg/mL), while higher concentrations did not contribute significantly to efficiency by forming wider inhibition zones. 

**Figure 1:**
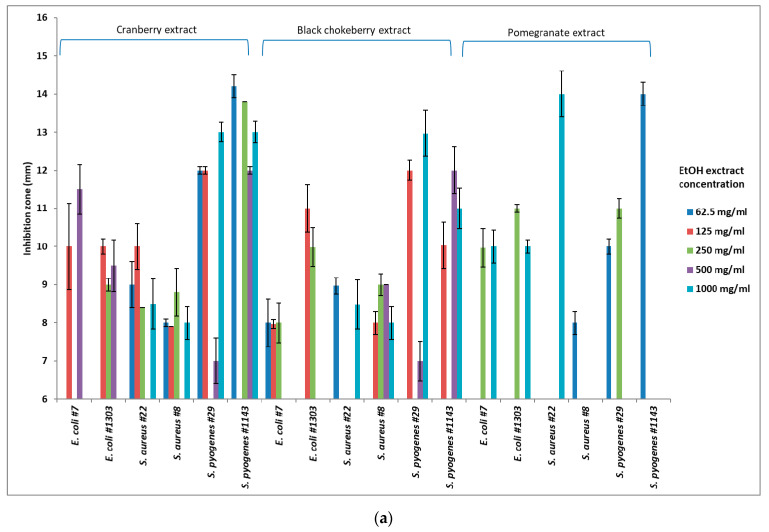

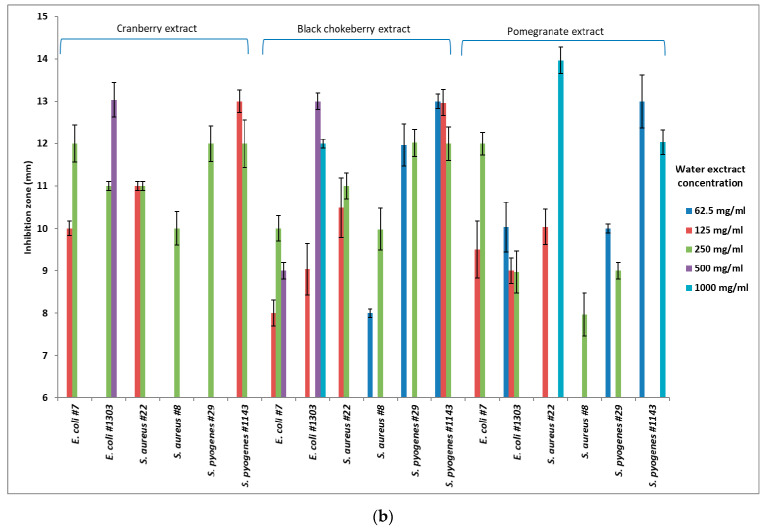
(**a**) Ethanolic extract concentration effectiveness (ZOI in mm ± SD) against different types of pathogens. (**b**) Aqueous extract concentration effectiveness (ZOI in mm ± SD) against different types of pathogens.

The above are supported by the corresponding statistical analysis, since, in the cranberry experiments, statistically significant positive correlations between the diameter of the inhibition zone and ethanolic extract concentration were observed for *E. coli* 7 (r = 0.66, *p* < 0.05) and *S. pyogenes* 1143 (r = 0.75, *p* < 0.05), as well as in the aqueous extract for *E. coli* 7 (r = 0.96, *p* < 0.05) and *E. coli* 1303 (r = 0.97, *p* < 0.05). Similarly, when pomegranate ethanolic extract was used, positive correlations were recorded for *S. aureus* 8 (r = 0.85, *p* < 0.05) and *S. pyogenes* (r = 0.90, *p* < 0.05), and in the case of aqueous extract from the same fruit for *E. coli* 7 (r = 0.91, *p* < 0.05) and *S. aureus* 22 (r = 0.98, *p* < 0.05). In black chokeberry experiments, there was an even lower correlation response given that the ZOI of only one strain positively correlated with the aqueous extract concentration (*S. aureus* 8, r = 0.92, *p* < 0.05). Data are graphically presented in Figure 1a for ethanolic extract and Figure 1b for aqueous extract.

### 3.7. Application of Aqueous Extracts in Raw Meatballs

From the application of aqueous extracts of the three fruits examined in a concentration of 2% *w/v*, some quite encouraging results for their antimicrobial activity were observed (Table 6).

**Figure 2:**
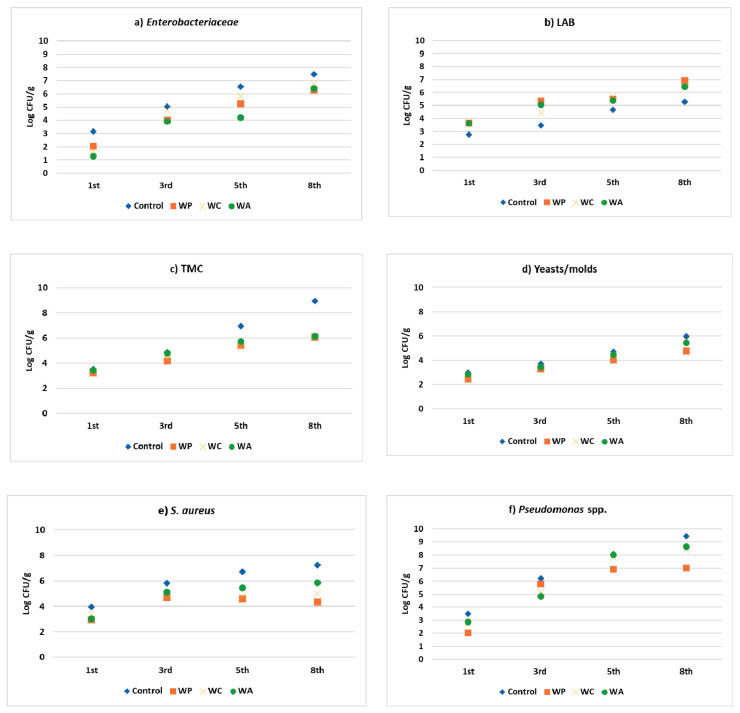
Viabilities (log CFU/g) of *Enterobacteriaceae* (**a**), lactic acid bacteria (LAB) (**b**), total mesophilic count (TMC) (**c**), yeasts/molds (**d**), *S. aureus* spp. (**e**), and *Pseudomonas* spp. (**f**) in raw meatball samples without aqueous extracts (C) and with black chokeberry (WA), pomegranate (WP), and cranberry (WC) aqueous extracts during the storage period.

As far as *Enterobacteriaceae* are concerned, our results showed a decrease in their growth if we compared the control samples with the respective aqueous extracts samples. The aqueous pomegranate extract especially exhibited the lowest growth rate by 1 log CFU/g lower on the eighth day. In our experiments, the samples with aqueous pomegranate extract provided a value of 4 log CFU/g on the third day of storage, and the remaining aqueous extracts showed a variation from 3.9 (black chokeberry) to 4.8 log CFU/g (cranberry) and 5.1 log CFU/g (control) on the third day of refrigerated storage (Figure 2a).

In the case of lactic acid bacteria (LAB), it was recorded that the values of the control samples varied from 2.8 to 5.2 log CFU/g, exhibiting the lowest microbial counts in comparison with all of the other samples containing plant extracts, the values of which ranged from 4 to approximately 7 log CFU/g. The highest value was noted in the case of samples with aqueous pomegranate extract (6.9 ± 1.4 log CFU/g) (Figure 2b).

Furthermore, in the case of total mesophilic count (TMC), the values revealed that the control samples surpassed the level of 6 log CFU/g, which is the limit of total viable counts in fresh ground meat according to European regulation (EC 2073/2005) [28], from the fifth day. Simultaneously, meat samples with aqueous extracts prolonged the date of their consumption until the eighth day. The samples with the aqueous pomegranate extract exhibited the lowest microbial count of 6.1 log CFU/g during the eighth day. This gives a time extension for which the product could be still available on the shelves (Figure 2c).

In addition, yeasts and molds exhibited a continual increase from 3 to 6 log CFU/g in the control samples from the beginning until the end of storage. The results for our meat samples were not very differentiated, with the only exception being the case of aqueous pomegranate extract, where the respective values of the count were from 2.4 to 4.8 log CFU/g until the end of storage (Figure 2d).

For *S. aureus*, the results of our assay revealed a range from 3.9 to 7.3 log CFU/g for our control in a period of eight days in storage at 4 °C. For our meat samples with aqueous pomegranate extracts, the respective count values were 3 to 4.3 log CFU/g for the same period. These results were the most promising in comparison to the meat samples with cranberry and black chokeberry extracts (5 and 5.9 log CFU/g, respectively, during the eighth day of storage) (Figure 2e).

Finally, in the case of *P. aeruginosa*, it was observed in our study that control samples showed microbial counts from 3.5 log CFU/g (1st day) to 9.5 log CFU/g (eighth day). The lowest values among the extract samples were observed in the case of pomegranate extract samples (2–7 log CFU/g in the respective days) (Figure 2f).

### 3.8. pH Values of Raw Meatballs during Refrigerated Storage

**Figure 3:**
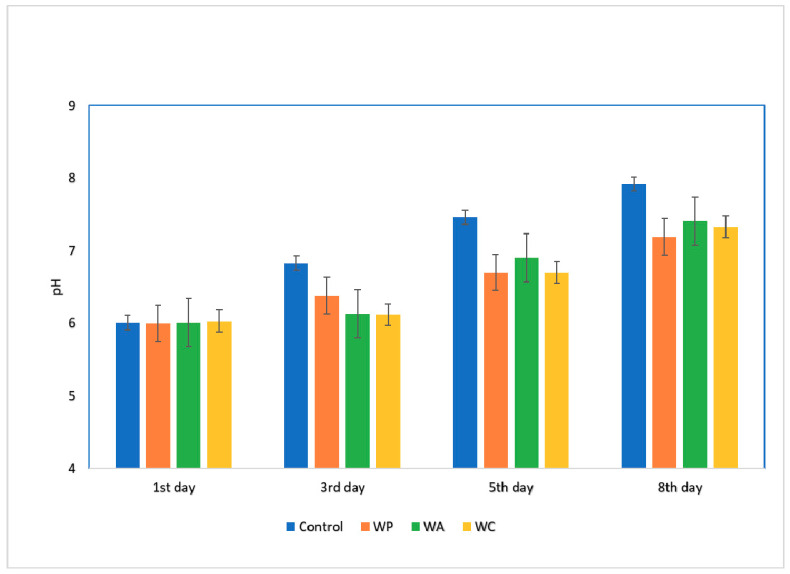
pH values during the period of refrigerated storage from the control and meatball samples with fruit extracts. Similar letters above bars indicate no significant interday differences (ANOVA, Tukey HSD) for each group of samples (control, WP: with aqueous pomegranate extract, WC: with aqueous cranberry extract, WC: with aqueous black chokeberry extract).

An extra note should be made about the role of pH during the entire period of storage, since pH reflects the rate of postmortem glycolysis (a key indicator of meat quality). Our pH values are shown in Figure 3. The initial value for all meat samples, including the control, was around 6.01. At the end of storage, the lowest pH value was measured in meatballs with aqueous pomegranate extract (7.19), followed by the samples with aqueous cranberry extracts (7.33), and, finally, samples with aqueous black chokeberry extract (7.41).

These outcomes indicate that all extracts incorporated in pork meatballs retarded the increase of pH values during refrigerated storage, compared to control samples (7.92). Similar studies verify our results [29]. This increasing pH level trend could be attributed to the high alkaline substance content end-up products from the degradation of proteins. The low pH values of samples with aqueous pomegranate extracts may explain the improved profile of these samples against foodborne pathogens and the elevated value of LAB viability in the same samples. Relatively high pH values of the samples may allow the dominance of *Pseudomonas* spp., which has been reported in several studies as the predominant spoiler in poultries stored under aerobic conditions [30].

## 4. Discussion

It is well-known that cranberries contain tannins, like proanthocyanidins, which are responsible for antimicrobial activity against a variety of bacteria [31].

The inhibitory effects against several foodborne pathogens have been established, e.g., *E. coli* and *S. aureus*, and attributed to the inhibition of extracellular microbial enzymes, deprivation of the substrates required for microbial growth, or even inhibition of oxidative phosphorylation [32,33].

Our results indicate that aqueous extracts of cranberry were the most effective against *E. coli*, *S. aureus*, and *S. pyogenes* strains. In the case of *E. coli* strains, this activity was concentration-dependent, since 5%, 10%, and 20% *v/v* of cranberry and pomegranate aqueous (*E. coli* 7) extracts exhibited ZOIs that became gradually larger. Similar results were observed for the *S. aureus* 8 strain in the case of aqueous cranberry and aqueous black chokeberry extracts.

Our results are in accordance with Tamkute et al. [18], who reported that an ethanol extract of cranberry demonstrated the strongest inhibitory effect against *S. aureus*, mainly by affecting peptidoglycan biosynthesis in bacteria cells. It was also observed that Gram-positive bacteria (*S. aureus* and *S. pyogenes* strains) exhibited larger ZOIs with lower extract concentrations in the respective solvents. This may be attributed to the differences between the cell walls, which affect osmotic cellular protection. More specifically, it has been suggested that berry phenolics may bind the outer cell wall membrane and disrupt the permeability barrier in Gram-negative bacteria, and thus, we can hypothesize that Gram-negative and Gram-positive bacteria undergo different types of damage from antimicrobial compounds [7]. In addition, different fractions of cranberry extracts have differentiated activity against Gram-negative and Gram-positive bacteria, for example, catechins, rhamnetin, and quercetin [34].

On the other hand, black chokeberry fruits (*Aronia melanocarpa*) are a rich source of anthocyanins, proanthocyanidins, and hydrocinnamic acids, as well as cranberry fruits [3]. Denev et al. [4] demonstrated that condensed tannins are the major antimicrobial agents of the berry. Their activity was tested against 10 human pathogens. *Proteus vulgaris* and *S. aureus* were the two more sensitive strains towards black chokeberry proanthocyanidins.

In the case of *E. coli*, we observed antimicrobial activity only with the application of water as the solvent in the case of cranberry and pomegranate extracts in a concentration-dependent or independent way. In contrast with Liepina et al. [32], we did observe antimicrobial activity by all of the extracts against *E. coli* strains. In their study, aqueous and ethanol extracts showed activity against *B. cereus*, *S. aureus*, and *P. aeruginosa* strains, but no activity against *E. coli* strains was observed. In our study, *S. aureus* strains were found to be more sensitive when tested with aqueous cranberry and black chokeberry extracts, but ethanol demonstrated better results when pomegranate extracts were examined. Penicillin gave a zone of inhibition with a diameter of 10–11 mm for the two *S. aureus* strains tested.

The results of the antimicrobial activity against *S. pyogenes* strains were quite promising. Particularly, aqueous extracts exhibited better results (ZOI ≥ 13 mm), not in a concentration-dependent way, in a low E.C. of 125 mg/mL (10% *v/v* solvent concentration). This can be explained by the exceptional water-soluble compounds, such as polysaccharides and polypeptides, that have inhibitory activity against pathogen adsorption [31]. In addition, anthocyanins are known for their antimicrobial potential against Gram-positive bacteria [34]. In the study of Liepina et al. [32], Gram-positive bacteria were found to be more susceptible to black chokeberry extracts, probably as an effect of the presence of anthocyanins.

As far as pomegranate extracts are concerned, their capacity to inhibit a variety of pathogenic bacteria has been assessed. Among the higher inhibitory effects of the examined methanol extract were found to be *E. coli* and *S. aureus* strains, with an MIC (minimum inhibitory concentration) of 50 mg/mL. These effects were mostly attributed to the presence of punicalagin in concentrations of 16.67 mg/g, as it was the most abundant phenolic compound in the methanol extract [16].

In another study, extracts using a mixture of water/ethanol or water/methanol/ethanol as solvents were prepared and tested against *Salmonella enterintidis* and *Salmonella kentucky* strains. The best results were recorded for water/methanol/ethanol and water/ethanol pomegranate peel extract. The tested concentrations varied from 1.56 to 200 mg/mL, and the inhibition zones had diameters of 22 mm and 22.2 mm for *Salmonella kentucky* and 19.8 mm and 18 mm for *Salmonella enterintidis*, respectively [21].

In our study, ethanol was an efficient solvent, exhibiting a wide ZOI at the lowest extract concentrations. Interestingly, in the case of *S. aureus* 22 and *S. pyogenes* 29 strains, the percentage of 5% *v/v* as the solvent concentration provided the most satisfying results. The same results can be observed when water is examined as the solvent in the case of *S. pyogenes* 1143 (cranberry and black chokeberry). This is in accordance with Prashanth et al. [35], who demonstrated that methanol extracts of pomegranate examined with the Microdilution Assay were more active against *S. aureus* (6 mg/mL) and *E. coli* (12 mg/mL) strains examined in comparison with their aqueous counterparts.

In contrast with McCarell et al. [36], who pointed out that aqueous extracts of pomegranate peels were not inhibitory to *E. coli* strains, our results indicate that aqueous extracts (20% *v/v*) reveal a ZOI with a diameter of 12 mm (an E.C. of 250 mg/mL).

Surprisingly, in *S. pyogenes* strains, it was observed that water (5%, 10%, and 20% *v/v*) and ethanol (5%, 10%, and 20% *v/v*) gave ZOIs equal or even larger than vancomycin (ZOI 10–12 mm), which was used as the positive control. This may be associated with the correlation between total phenolics and antimicrobial activity of *Punica granatum*, since metabolic toxins of broad-spectrum antibiotic compounds are present in pomegranate peels [37].

Polyphenols are hydrophilic components, and thus, more hydrophilic extractants can be used as better solvents for their extraction [38]. Their sulfhydryl groups of proteins [14] are responsible for their antimicrobial properties. Pomegranate extracts are able to inhibit the production of bacterial protein secretions, and thus the production of enterotoxins [39]. It should be noted that the mechanism of action of phenolic compounds is also based on the high reactivity of their hydroxyl groups, especially under aqueous conditions, which lead to the deformation and retardation of bacterial growth. They are also involved in the inactivation of bacterial enzymes, cell wall binding, and intercalation into the bacterial DNA during replication [40].

Gram-positive bacteria are considered more sensitive than Gram-negative bacteria against plant extracts, mostly because of differences in the structure of their cell walls. Our study confirmed this as well. *E. coli* strains recorded smaller ZOIs in both solvents in comparison to *S. aureus* and *S. pyogenes* strains, which are Gram-positive bacteria. Apparently, many pomegranate peel phytochemicals, like ellagic and gallic acids, are implicated in the mechanism of antibacterial action of the plant.

In total, as far as the achieved ZOIs among all of the extracts applied are concerned, it can be stated that aqueous and ethanolic extracts are effective against all types of pathogens examined. This makes the results even more promising, since ethanol and water are two solvents that are suitable for application to food products.

Indeed, the application of aqueous extracts of the three examined fruits in pork meatballs led to very satisfying results regarding antimicrobial activity. Specifically, the best results were achieved in the case of aqueous extract from pomegranate, since the respectively produced meat balls were preserved three more days compared to the control samples, as far as total mesophilic count is concerned. Additionally, the levels of all of the examined bacterial groups (*Enterobacteriaceae*, yeasts/molds, *Pseudomonas* spp., *Staplylococcus* spp.) decreased in the meatball samples that contained aqueous extracts of all three fruits for the entire storage period. As far as *Enterobacteriaceae* are concerned, Sojic’s study [41] demonstrated that the respective rate of growth varied from 3.2 (control) to 3.5 log CFU/g (meat samples with 0.075 μL/g of supercritical fluid thyme extract).

In the case of total mesophilic counts, in comparison with other studies [42,43], the addition of cauliflower leaf extracts and super critical fluid wild thyme extracts (SFEs) in ground pork patties showed that TMC reached the value of 6.17 log CFU/g on the fifth day and the value of 6.82 log CFU/g the third day of storage, respectively.

For LAB behavior, Ranucci et al. [42] applied a mix of *Punica granatum* and *Citrus* spp. extracts in two different concentrations (5% and 10%) and examined the total viable count (TVC), LAB, and psychrotrophic microbial count. Lower values of microbial loads were recorded in the two mixes *Punica granatum* of the two different concentrations in comparison with control samples. On the contrary, Lytou et. al. [43] examined the impact of pomegranate juice marinades on marinated chicken samples. Their findings are in complete accordance with our results in approximately all of the microbial groups tested (TVC, *Pseudomonas* spp., *Enterobacteriaceae*, LAB, yeasts/molds). This fact may be explained by the decreased pH values, which appeared in the samples containing extracts, compared to control samples, as it is well-known that low pH values favor LAB development.

In the case of *S. aureus*, our results are in accordance with Guo’s study [44], where *Amaranthus tricolor* crude extract was applied in cooked pork meat. The count values in their study ranged from 3 to 4 log CFU/g for the control and 3 to 2.5 log CFU/g for their meat samples, including extracts.

For yeasts/molds, in another assay [45], approximately the same results were recorded when they examined the impact of the addition of extracts from *Carissa carandas* fruits in ground pork during storage at 4 °C. There, the yeasts’ microbial count ranged from 4.03 to 5.16 log CFU/g for the control and meat samples as well. 

Finally, for *P. aeruginosa*, Moorthy et al. [46] tested a total of 19 bacteria by a disk diffusion method and broth dilution method versus ethanolic pericarp extract of pomegranate. The results revealed that significant bactericidal activity against *S. aureus* (19.2 mm), *E. coli* (18.4 mm), and *P. aeruginosa* (18.2 mm) was recorded. This fact is in accordance with our study. Sadeghian et al. [47] applied aqueous pomegranate extracts and reported strong antimicrobial activity against *S. aureus* and *P. aeruginosa* strains. The antimicrobial activity of both extracts was comparable with those of cloxacillin, gentamycin, and clotrimazole.

Interestingly, LAB viability was increased in our extract samples, a fact that is desirable given the numerous advantages that these bacteria provide to the host [48].

## 5. Conclusions

The antimicrobial effectiveness of cranberry, black chokeberry, and pomegranate extracts against food pathogens led to encouraging results. Our promising results indicate that both ethanol and water as solvents in low concentrations and their respective extracts even in low concentrations can be highly effective against the foodborne pathogens examined. Further investigation and application of aqueous extracts in raw pork meatballs took place in order to evaluate their technological potential. The aqueous extracts improved the microbiological profile of the meatball samples compared to control samples. It can be concluded that all extracts were almost equally effective. In conclusion, the examined extracts could be applied to meat products as additives for shelf life prolongation and ameliorated antimicrobial activities. Nevertheless, additional studies should be done concerning not only the prevalence of the benefits or the viability of spoilage/pathogenic bacteria, but also on the organoleptic and physicochemical characteristics of the foods containing these or analogous plant extracts.

## Figures and Tables

**Table 1:** Zones of inhibition (ZOIs) of cranberry extract against *Escherichia coli*, *Staphylococcus aureus*, and *Streptococcus pyogenes*.

Solvents	Concentration of Solvents		*E. coli*		*S. aureus*		*S. pyogenes*	
			7	1303	22	8	29	1143
EtOH	5%	Diameter of ZOI in mm	11 ± 0.4 ^b^	9 ± 0.2 ^a^	9 ± 0.6 ^a^	9 ± 0.6 ^a^	12 ± 0.1 ^c^	14 ± 0.3 ^d^
		Extract Concentration (mg/mL)	125	250	62.5	250	125	62.5
	10%	Diameter of ZOI in mm	12 ± 0.5 ^a^	10 ± 0.2 ^b^	9 ± 0.3 ^b^	8 ± 0.1 ^d^	7 ± 0.6 ^c^	12 ± 0.1 ^a^
		Extract Concentration (mg/mL)	500	500	1000	125	500	500
	20%	Diameter of ZOI in mm	11 ± 0.2 ^c^	9 ± 0.6 ^b^	8 ± 0.5 ^a^	8 ± 0.4 ^a^	13 ± 0.3 ^d^	13 ± 0.3 ^d^
		Extract Concentration (mg/mL)	500	500	1000	1000	1000	1000
Water	5%	Diameter of ZOI in mm	9 ± 0.1 ^b^	10 ± 0.2 ^c^	10 ± 0.6 ^c^	8 ± 0.1 ^a^	12 ± 0.1 ^d^	14 ± 0.4 ^e^
		Extract Concentration (mg/mL)	125	125	125	62.5	62.5	62.5
	10%	Diameter of ZOI in mm	10 ± 0.2 ^a^	11 ± 0.1 ^b^	11 ± 0.1 ^b^	10 ± 0.1 ^a^	12 ± 0.6 ^c^	13 ± 0.3 ^d^
		Extract Concentration (mg/mL)	125	250	125	250	250	125
	20%	Diameter of ZOI in mm	12 ± 0.4 ^c^	13 ± 0.4 ^d^	11 ± 0.1 ^b^	10 ± 0.6 ^a^	12 ± 0.3 ^c^	12 ± 0.6 ^c^
		Extract Concentration (mg/mL)	250	500	250	250	250	250
Penicillin			-	-	12 ± 1.7	10.0 ± 0.0	-	-
Levofloxacin			32.3 ± 0.6	31.7 ± 2.9	-	-	-	-
Vancomycin			-	-	-	-	10.7 ± 0.6	14 ± 1.7

Different superscript letters in a row indicate statistically significant differences (ANOVA, LSD).

**Table 2:** Zones of inhibition (ZOIs) of black chokeberry extract against *Escherichia coli*, *Staphylococcus aureus*, and *Streptococcus pyogenes*.

Solvents	Concentration of Solvents		*E. coli*		*S. aureus*		*S. pyogenes*	
			7	1303	22	8	29	1143
EtOH	5%	Diameter of ZOI in mm	8 ± 0.6 ^a^	11 ± 0.6 ^d^	9 ± 0.2 ^b^	9 ± 0.2 ^b^	12 ± 0.3 ^e^	10 ± 0.6 ^c^
		Extract Concentration (mg/mL)	62.5	125	62.5	250	125	125
	10%	Diameter of ZOI in mm	8 ± 0.1 ^b^	10 ± 0.6 ^d^	9 ± 0.3 ^c^	8 ± 0.3 ^b^	7 ± 0.5 ^a^	12 ± 0.6 ^e^
		Extract Concentration (mg/mL)	125	250	1000	125	500	500
	20%	Diameter of ZOI in mm	8 ± 0.5 ^a^	10 ± 0.5 ^b^	8 ± 0.4 ^a^	8 ± 0.4 ^a^	13 ± 0.6 ^d^	11 ± 0.5 ^c^
		Extract Concentration (mg/mL)	250	250	1000	1000	1000	1000
Water	5%	Diameter of ZOI in mm	8 ± 0.3 ^a^	9 ± 0.6 ^b^	10 ± 0.5 ^c^	8 ± 0.1 ^a^	12 ± 0.5 ^d^	13 ± 0.2 ^e^
		Extract Concentration (mg/mL)	125	125	125	62.5	62.5	62.5
	10%	Diameter of ZOI in mm	10 ± 0.3 ^a^	13 ± 0.2 ^d^	11 ± 0.5 ^b^	10 ± 0.6 ^a^	12 ± 0.4 ^c^	13 ± 0.3 ^d^
		Extract Concentration (mg/mL)	250	500	125	250	250	125
	20%	Diameter of ZOI in mm	9 ± 0.2 ^a^	12 ± 0.1 ^d^	11 ± 0.3 ^c^	10 ± 0.5 ^b^	12 ± 0.3 ^d^	12 ± 0.4 ^d^
		Extract Concentration (mg/mL)	500	1000	250	250	250	250
Penicillin			-	-	12 ± 1.7	10.0 ± 0.0	-	-
Levofloxacin			32.3 ± 0.6	31.7 ± 2.9	-	-	-	-
Vancomycin			-	-	-	-	10.7 ± 0.6	15 ± 1.7

Different superscript letters in a row indicate statistically significant differences (ANOVA, LSD).

**Table 3:** Zones of inhibition (ZOIs) of pomegranate extract against *Escherichia coli*, *Staphylococcus aureus*, and *Streptococcus pyogenes*.

Solvents	Concentration of Solvents		*E. coli*		*S. aureus*		*S. pyogenes*	
			7	1303	22	8	29	1143
EtOH	5%	Diameter of ZOI in mm	10 ± 0.3 ^b^	11 ± 0.1 ^c^	15 ± 0.2 ^e^	8 ± 0.3 ^a^	10 ± 0.2 ^b^	14 ± 0.3 ^d^
		Extract Concentration (mg/mL)	500	250	500	62.5	62.5	62.5
	10%	Diameter of ZOI in mm	10 ± 0.4 ^a^	10 ± 0.2 ^a^	10 ± 0.4 ^a^	12 ± 0.1 ^c^	11 ± 0.1 ^b^	12 ± 0.6 ^c^
		Extract Concentration (mg/mL)	1000	1000	500	500	250	500
	20%	Diameter of ZOI in mm	10 ± 0.5 ^a^	11 ± 0.2 ^b^	14 ± 0.6 ^d^	10 ± 0.2 ^a^	11 ± 0.4 ^b^	12 ± 0.3 ^c^
		Extract Concentration (mg/mL)	250	500	1000	500	250	500
Water	5%	Diameter of ZOI in mm	9 ± 0.1 ^a^	10 ± 0.6 ^b^	12 ± 0.2 ^c^	8 ± 0.5 ^a^	10 ± 0.1 ^b^	13 ± 0.6 ^d^
		Extract Concentration (mg/mL)	125	62.5	500	250	62.5	62.5
	10%	Diameter of ZOI in mm	10 ± 0.6 ^c^	9 ± 0.3 ^b^	10 ± 0.4 ^c^	8 ± 0.3 ^a^	9 ± 0.2 ^b^	11 ± 0.6 ^d^
		Extract Concentration (mg/mL)	125	125	125	500	250	500
	20%	Diameter of ZOI in mm	12 ± 0.3 ^d^	9 ± 0.5 ^b^	14 ± 0.3 ^c^	8 ± 0.1 ^a^	11 ± 0.6 ^c^	12 ± 0.3 ^d^
		Extract Concentration (mg/mL)	250	250	1000	500	500	1000
Penicillin			-	-	12 ± 1.7	10.0 ± 0.0	-	-
Levofloxacin			32.3 ± 0.6	31.7 ± 2.9	-	-	-	-
Vancomycin			-	-	-	-	10.7 ± 0.6	14 ± 1.7

Different superscript letters in a row indicate statistically significant differences (ANOVA, LSD).

**Table 4:** Zones of inhibition (ZOIs) of various fruit extracts against pathogens.

Extract	Strain	EtOH	Water	*ANOVA (F,p)*
Cranberry	*E. coli* 7	11.3 ± 0.6 ^a,2^	10.3 ± 1.3 ^a,2^	*4.14, p > 0.05*
	*E. coli* 1303	9.3 ± 0.6 ^a,1^	11.3 ± 1.3 ^b,3,4^	*16.51, p < 0.05*
	*S. aureus* 22	8.7 ± 0.6 ^a,1^	10.7 ± 0.6 ^b,2,3^	*46.13, p < 0.05*
	*S. aureus* 8	8.3 ± 0.6 ^a,1^	9.3 ± 1.0 ^b,1^	*6.20, p < 0.05*
	*S. pyogenes* 29	10.7 ± 2.8 ^a,2^	12.0 ± 0.3 ^a,4^	*2.00, p > 0.05*
	*S. pyogenes* 1143	13.0 ± 0.9 ^a,3^	13.0 ± 0.9 ^a,5^	*0.01, p > 0.05*
	*ANOVA (F,p)*	*17.15, p < 0.05*	*14.83, p < 0.05*	
Black chokeberry	*E. coli* 7	8.0 ± 0.4 ^a,1^	9.0 ± 0.9 ^d,2^	*9.45, p < 0.05*
	*E. coli* 1303	10.3 ± 0.7 ^a,2^	11.3 ± 1.8 ^a,3,4^	*2.47, p > 0.05*
	*S. aureus* 22	8.6 ± 0.6 ^a,1^	10.6 ± 0.6 ^b,2,3^	*50.17, p < 0.05*
	*S. aureus* 8	8.3 ± 0.6 ^a,1^	9.3 ± 1.06 ^b,1^	*5.99, p < 0.05*
	*S. pyogenes* 29	10.7 ± 2.8 ^a,2^	12.0 ± 0.3 ^a,4^	*2.04, p > 0.05*
	*S. pyogenes* 1143	11.0 ± 1.0 ^a,2^	12.6 ± 0.6 ^b,5^	*18.81, p < 0.05*
	*ANOVA (F,p)*	*9.19, p < 0.05*	*18.88, p < 0.05*	
Pomegranate	*E. coli* 7	9.98 ± 0.4 ^a,1^	10.3 ± 1.4 ^a,2^	0.54, *p* > 0.05
	*E. coli* 1303	10.7 ± 0.5 ^a,1^	9.3 ± 0.7 ^b,2^	22.38, *p* < 0.05
	*S. aureus* 22	13.0 ± 2.3 ^a,2^	12.0 ± 1.7 ^a,3^	1.08, *p* > 0.05
	*S. aureus* 8	9.9 ± 1.7 ^a,1^	7.9 ± 0.3 ^b,1^	11.64, *p* < 0.05
	*S. pyogenes* 29	10.7 ± 0.5 ^a,1^	10.0 ± 0.9 ^a,2^	3.27, *p* > 0.05
	*S. pyogenes* 1143	12.7 ± 1.0 ^a,2^	12.0 ± 0.9 ^a,3^	1.92, *p* > 0.05
	*(ANOVA F, p)*	*9.40, p < 0.05*	*18.28, p < 0.05*	

Different superscript letters in a row or numbers in column indicate statistically significant differences (ANOVA, LSD).

**Table 5:** Pearson correlation coefficients between solvent concentration (5%, 10%, and 20%) and effectiveness (zone of inhibition in mm) against pathogens for the various extracts.

Extract	Strain	EtOH	Water
Cranberry	*E. coli* 7	ns	0.98 **
	*E. coli* 1303	ns	0.98 **
	*S. aureus* 22	−0.72 *	ns
	*S. aureus* 8	ns	0.72 *
	*S. pyogenes* 29	ns	ns
	*S. pyogeness* 1143	ns	−0.90 **
*Black chokeberry*	*E. coli* 7	ns	ns
	*E. coli* 1303	ns	ns
	*S. aureus* 22	−0.83 **	ns
	*S. aureus* 8		0.69 *
	*S. pyogenes* 29	ns	ns
	*S. pyogeness* 1143	ns	−0.84 **
Pomegranate	*E. coli* 7	ns	0.97 **
	*E. coli* 1303	ns	ns
	*S. aureus* 22	ns	ns
	*S. aureus* 8	ns	ns
	*S. pyogenes* 29	−0.69 *	ns
	*S. pyogeness* 1143	ns	ns

Pearson product moment correlation between each pair of variables, * *p* < 0.05, ** *p* < 0.005, ns = not significant.

**Table 6:** Bacterial populations of raw meatballs during the period of cold storage according to the aqueous extract used as a preservative (WP: pomegranate, WC: cranberry, WC: black chokeberry).

Species	Sample	First Day	Third Day	Fifth Day	Eighth Day
*Enterobacteriaceae*	Control	3.1 ± 0.8 ^a1^	5.1 ± 1.1 ^ab1^	6.5 ± 1.4 ^ab1^	7.5 ± 1.8 ^b1^
	WP	2.0 ± 0.8 ^a12^	4.0 ± 0.7 ^ab1^	5.2 ± 1.1 ^b1^	6.3 ± 1.1 ^b1^
	WC	1.7 ± 0.4 ^a12^	4.8 ± 1.1 ^b1^	5.8 ± 1.4 ^b1^	6.8 ± 1.1 ^b1^
	WA	1.3 ± 0.2 ^a2^	3.9 ± 1.4 ^ab1^	4.2 ± 1.2 ^ab1^	6.4 ± 1.7 ^b1^
LABs	Control	2.8 ± 0.3 ^a1^	3.4 ± 0.8 ^ab1^	4.7 ± 0.8 ^ab1^	5.2 ± 1.1 ^b1^
	WP	3.6 ± 0.3 ^a2^	5.3 ± 0.9 ^ab1^	5.5 ± 1 ^ab1^	6.9 ± 1.4 ^b1^
	WC	3.6 ± 0.1 ^a2^	5.0 ± 1.3 ^a1^	5.6 ± 2 ^a1^	6.5 ± 2.3 ^a1^
	WA	3.6 ± 0.4 ^a2^	5.0 ± 1.4 ^a1^	5.6 ± 2.1 ^a1^	6.5 ± 2.4 ^a1^
TMC	Control	3.5 ± 0.3 ^a1^	7.9 ± 1.1 ^b1^	6.9 ± 1.5 ^b1^	9.0 ± 1.5 ^b1^
	WP	3.2 ± 0.5 ^a1^	4.2 ± 1.4 ^a2^	5.4 ± 1.2 ^a1^	6.1 ± 2.9 ^a1^
	WC	3.2 ± 0.3 ^a1^	4.3 ± 1.3 ^a12^	5.3 ± 2.5 ^a1^	6.2 ± 1.5 ^a1^
	WA	3.4 ± 0.4 ^a1^	4.8 ± 1.8 ^a12^	5.4 ± 1.5 ^a1^	6.2 ± 2.4 ^a1^
Yeast/Molds	Control	3.0 ± 0.6 ^a1^	3.7 ± 1.7 ^a1^	4.7 ± 1.6 ^a1^	6.0 ± 2.5 ^a1^
	WP	2.4 ± 0.3 ^a1^	3.3 ± 1.5 ^a1^	4.0 ± 1.3 ^a1^	4.8 ± 2.4 ^a1^
	WC	2.5 ± 0.7 ^a1^	3.4 ± 1.6 ^a1^	4.4 ± 1.8 ^a1^	5.6 ± 2.2 ^a1^
	WA	2.8 ± 0.4 ^a1^	3.5 ± 1.6 ^a1^	4.5 ± 2.3 ^a1^	5.4 ± 1.8 ^a1^
*Staphylococcus* spp	Control	3.9 ± 0.7 ^a1^	5.8 ± 1.8 ^a1^	6.7 ± 1.5 ^a1^	7.3 ± 1.5 ^a1^
	WP	2.9 ± 0.3 ^a1^	4.7 ± 1.2 ^a1^	4.6 ± 1.7 ^a1^	4.3 ± 2.2 ^a1^
	WC	3.4 ± 0.3 ^a1^	5 ± 1.6 ^a1^	4.8 ± 1.8 ^a1^	5.0 ± 1.7 ^a1^
	WA	3.0 ± 0.4 ^a1^	5.1 ± 1.6 ^a1^	5.5 ± 1.4 ^a1^	5.9 ± 1.5 ^a1^
*Pseudomonas* spp	Control	3.5 ± 0.5 ^a1^	6.2 ± 1.1 ^ab1^	8.1 ± 1.7 ^b1^	9.5 ± 1.6 ^b1^
	WP	2.0 ± 0.3 ^a2^	5.8 ± 1.4 ^b1^	6.9 ± 0.2 ^b1^	7.0 ± 0.9 ^b1^
	WC	2.8 ± 0.5 ^a12^	5.2 ± 1.4 ^ab1^	7.8 ± 2.4 ^b1^	8.4 ± 1.5 ^b1^
	WA	2.9 ± 0.5 ^a12^	4.8 ± 1.8 ^ab1^	8.0 ± 1.1 ^bc1^	8.6 ± 1.3 ^c1^

Similar superscript letters in rows indicate no significant differences (ANOVA, Tukey HSD). Similar superscript numbers in columns indicate no significant differences (ANOVA, Tukey HSD) among the bacterial species.

## Data Availability

Main data are contained within the article. Further data presented in this study are available upon reasonable request from the corresponding author.
